# Physicians’ Perceptions of AI and Extended Reality in Telemedicine: A Multi-Specialty Cross-Sectional Survey in Romania

**DOI:** 10.3390/healthcare13212675

**Published:** 2025-10-23

**Authors:** Florina Onetiu, Melania Lavinia Bratu, Felix Bratosin, Tiberiu Bratu

**Affiliations:** 1 Doctoral School, “Victor Babes” University of Medicine and Pharmacy, Eftimie Murgu Square 2, 300041 Timisoara, Romania; florina.onetiu@umft.ro; 2 Department of Neurosciences, “Victor Babes” University of Medicine and Pharmacy, Eftimie Murgu Square 2, 300041 Timisoara, Romania; 3 Multidisciplinary Research Center for Infectious Diseases, “Victor Babes” University of Medicine and Pharmacy, Eftimie Murgu Square 2, 300041 Timisoara, Romania; felix.bratosin@umft.ro; 4 Discipline of Plastic Surgery, “Victor Babes” University of Medicine and Pharmacy, Eftimie Murgu Square 2, 300041 Timisoara, Romania; tiberiu.bratu@umft.ro

**Keywords:** telemedicine, extended reality, artificial intelligence, technology acceptance, UTAUT, Romania, health policy, medical education

## Abstract

**Background and Objectives:** Artificial intelligence (AI) and extended reality (XR) are reshaping telemedicine, yet physician-level adoption depends on perceived value, training needs, and specialty context. We quantified attitudes toward AI/XR, identified barriers/benefits, and tested advanced relationships (moderation and mediation). **Methods:** Cross-sectional survey of Romanian physicians (*n* = 43) across anesthesiology and ICU, surgical, medical, and dentistry. Items were translated into English and mapped to 5-point scales. A 10-item Telemedicine Acceptance Index (TAI; α = 0.86) and a 2-item XR Utility Index (XUI) were computed. Moderation by specialty (Training Priority × Specialty) and bootstrap mediation (2000 resamples) of Future Potential → XUI → TAI were performed. **Results:** Overall acceptance and perceived utility of XR were moderate to high across specialties; participants most frequently identified technical and financial constraints as barriers and time efficiency and improved access as key benefits. Acceptance patterns were similar across specialties and aligned most strongly with beliefs about future system-level potential and the priority assigned to hands-on training. **Conclusions:** Physicians reported favorable acceptance of AI/XR-enabled telemedicine. Perceived future system-level value and prioritization of hands-on training were the most consistent correlates of acceptance across specialties. Technical and financial constraints remained the primary barriers, while time efficiency and access emerged as leading perceived benefits. Acceptance appears to be driven more by beliefs about system-level potential and practical upskilling than by specialty identity.

## 1. Introduction

Telemedicine has moved from contingency adoption during the COVID-19 pandemic to an embedded mode of care with maturing clinical, technical, and organizational frameworks. Pre-pandemic overviews positioned telehealth as a long-term restructuring of delivery [1]. At the same time, early-pandemic surveillance documented a more than 150% surge in U.S. telehealth utilization across weeks 10–13 of 2020, catalyzed by policy, reimbursement, and workflow adjustments [2]. Beyond volume metrics, contemporary meta-analyses indicate that telemedicine can deliver clinically meaningful outcomes in chronic cardiovascular care, reducing cardiovascular hospitalizations and mortality relative to usual care in pooled analyses, suggesting that the modality has evolved from an “access extender” to an outcome-relevant intervention for selected conditions [3]. Yet randomized evidence is heterogeneous across settings: the TELESCOPE trial found no reduction in ICU length of stay with a rigorously implemented tele-ICU program, underscoring that technology alone is insufficient without complementary process redesign [4].

Against this backdrop, clinician acceptance remains a principal rate-limiting step for value realization. Within health informatics, the Technology Acceptance Model (TAM) and related frameworks have consistently linked perceived usefulness and ease-of-use to behavioral intention among clinicians [5], and multi-country surveys continue to map structural and attitudinal facilitators/barriers for telemedical consultations, such as workload fit, medico-legal clarity, and infrastructure reliability [6]. Country-level context also matters: in Romania, a nationwide TAM analysis of >1000 physicians reported that perceived usefulness and subjective norms were strong, independent predictors of intention to use telemedicine, with accessibility to medical records and incentives further shaping adoption [7]. These constructs guided our a priori hypothesis that higher training priority and stronger beliefs in system-level potential would predict acceptance in our sample.

Concurrently, AI capabilities increasingly undergird telemedicine workflows, triage, risk stratification, decision support, and documentation, while XR (augmented, mixed, virtual reality) augments remote presence, telementoring, and procedural guidance. Authoritative reviews characterize machine learning’s clinical promise and translational hurdles (data quality/shift, calibration, deployment) [8]. But acceptance is tempered by well-documented risks: algorithmic bias can systematically misallocate resources (e.g., a widely used U.S. risk tool under-referred sicker Black patients due to cost-as-proxy design) [9]; and leading scholars argue current “explainability” techniques often fall short of supporting bedside decisions, calling instead for rigorous internal/external validation pipelines to earn trust [10]. These issues, fairness, transparency, and accountability, are not abstract; they shape whether practicing physicians will endorse and rely on AI-enabled telemedicine.

Security, privacy, and resilience further contour acceptance. Clinicians are increasingly identifying cybersecurity as integral to patient safety and organizational continuity, citing workflow disruptions, insider threats, and legacy systems as salient barriers to digital care at scale [11]. Specialty-specific XR evidence is also maturing: Randomized trials show that augmented-reality (AR) telementoring and telestration can improve task performance and safety in minimally invasive surgery training, supporting the plausibility of XR-enabled remote guidance in real clinical environments [12,13]. Systematic reviews synthesize real-time AR telemedicine/telementoring implementations, identifying usability, ergonomics, and network stability as adoption determinants—considerations likely to be salient to physicians evaluating XR utility in everyday workflows [14].

Beyond surgery, dentistry, and other ambulatory domains, there is a growing use of tele-examination, asynchronous image exchange, and digital tools that can be paired with AI decision support and XR visualization. Reviews point to advantages in access and efficiency, alongside requirements for training and adherence to standards [15]. Notably, acceptance is not monolithic across clinical communities: even within the same health system, determinants vary by workload, medico-legal exposure, and perceived task-technology fit. Studies in UTAUT/TAM traditions show that performance expectancy, facilitating conditions, and digital literacy are recurrent predictors among health professionals, aligning with our focus on training priority as a modifiable lever [5,6,7].

Taken together, the scholarship implies that Romanian physicians’ attitudes toward AI/XR-enabled telemedicine will be shaped by (i) demonstrated clinical utility in their domain; (ii) credible mitigations for bias, explainability limits, and security risks; and (iii) pragmatic supports—training, integration with records, and clear governance. Our cross-sectional, multi-specialty survey therefore quantifies acceptance and perceived utility, characterizes benefits and barriers, compares specialty clusters, and models predictors with a priori emphasis on training and beliefs about system-level potential. By centering practicing clinicians rather than students or patients, the study aims to inform targeted implementation strategies that are sensitive to Romania’s mixed public–private ecosystem and uneven digital infrastructure [7,11,12,13].

Consistent with Technology Acceptance Model and UTAUT frameworks [14], Perceived Future Potential reflects performance expectancy (anticipated gains in care quality/efficiency at system level), Training Priority embodies facilitating conditions and effort expectancy via planned capability building and workflow support, and the XR Utility Index (XUI) captures task–technology fit for immersive visualization and telementoring. Our modeling, therefore, estimates how performance expectancy and facilitating conditions independently relate to acceptance and whether task-specific XR utility transmits (mediates) part of that influence.

Romania combines a mixed public–private delivery model [15], uneven digital infrastructure across regions, and rapid alignment with EU digital and AI regulatory frameworks. This mix creates both high perceived utility (access/efficiency) and salient constraints (connectivity, financing, data protection). Studying physician acceptance under these conditions offers implementation-relevant insight for similar health systems, while acknowledging that generalizability beyond comparable contexts requires caution.

Recent clinician surveys across Europe, North America, and Asia have identified performance expectancy, facilitating conditions, medico-legal clarity, and training as recurrent determinants of telemedicine adoption; XR-focused syntheses highlight ergonomics and reliability as adoption prerequisites [16,17].

This study aimed to quantify physician acceptance of AI/XR-enabled telemedicine in Romania, identify modifiable drivers of acceptance, and probe specialty-specific patterns. Consistent with TAM/UTAUT, we pre-registered four hypotheses: H1, higher Perceived Future Potential associates with higher acceptance (TAI); H2, higher Training Priority associates with higher TAI; H3, XR Utility (XUI) partially mediates the Perceived-Future-Potential and TAI association. Beyond descriptive attitudes, this study aims to model performance expectancy (Perceived Future Potential) and facilitating conditions (Training Priority), test specialty moderation of the training–acceptance link, and probe mediation by XR task-utility (XUI)—a combined framework that, to our knowledge, has not been simultaneously examined in a Romanian multi-specialty clinician sample

## 2. Materials and Methods

### 2.1. Study Design, Setting, and Ethics

We employed pragmatic, non-probability sampling to enroll licensed physicians currently practicing in Romania. Recruitment targeted four a priori specialty clusters (A&ICU, Surgical, Medical, Dentistry) to capture workflow diversity; larger enrollment in A&ICU reflects local staffing patterns and the service’s high exposure to technology-mediated workflows. Participation was voluntary and uncompensated. No patient data or identifiable personal data were collected; the exported analytical file contained only survey responses and specialty categories. The project was conceived for educational/research training purposes and conducted in accordance with the Declaration of Helsinki.

In this study, telemedicine denotes synchronous/asynchronous clinical encounters and decision support (triage, documentation, remote consultation). Tele-monitoring—remote physiologic surveillance—is a related but distinct service and was not the focus of sampling.

The Local Commission of Ethics for Scientific Research the Victor Babes University of Medicine and Pharmacy from Timisoara, Romania, operates under article 167 provisions of Law no. 95/2006, art. 28, chapter VIII of order 904/2006; with EU GCP Directives 2005/28/EC, International Conference of Harmonization of Technical Requirements for Registration of Pharmaceuticals for Human Use (ICH). The study was approved by the Ethics Committee of Victor Babes University of Medicine and Pharmacy (protocol code 2429 and date of approval: 10 January 2023).

### 2.2. Participants and Recruitment

We employed pragmatic, non-probability sampling to enroll licensed physicians practicing in Romania (age ≥ 18 years). Recruitment targeted four a priori specialty clusters (A&ICU, Surgical, Medical, Dentistry) to capture workflow diversity; larger enrollment in A&ICU reflects local staffing patterns and the service’s high exposure to technology-mediated workflows. The final analytic sample comprised *n* = 43 complete respondents. Given the exploratory, training-focused scope and uneven specialty strata in routine practice, we prioritized content coverage over formal sample-size powering. A post hoc sensitivity check indicates that with *n* = 43, we had ~80% power (α = 0.05, two-sided) to detect correlations of |ρ| ≈ 0.40 or larger, while between-group differences smaller than moderate effects were likely underpowered; non-significant findings in group tests are interpreted accordingly.

### 2.3. Instrument and Translation

The 21-item instrument was drafted from TAM/UTAUT constructs and telemedicine/XR adoption in the literature, then reviewed by two clinicians and one methodologist for face/content validity. All items used 5-point Likert-type scales oriented so higher values indicated more favorable perceptions. The Telemedicine Acceptance Index (TAI) averaged 10 items (care quality, efficiency, diagnostic support, patient engagement, error reduction, recommendation frequency, treatment personalization, decision support, pandemic usefulness, patient receptivity; Cronbach’s α = 0.86). XR Utility Index (XUI) averaged two items (simulation/training and complex procedures). Single-item predictors captured training priority, perceived future potential, and curricular integration.

Physicians’ attitudes toward AI-enabled telemedicine were captured with a 10-item Telemedicine Attitudes Index (TAI; averaged score), which showed good internal consistency (Cronbach’s α = 0.86; *n* = 43), with factor-analytic validation (EFA/CFA) deferred to future, larger samples. The complete English TAI items are: (1) AI-enabled telemedicine can improve overall care quality; (2) can improve care efficiency; (3) improves diagnostic support; (4) improves patient engagement; (5) can reduce errors; (6) I would recommend AI-enabled telemedicine to appropriate patients; (7) supports treatment personalization; (8) AI decision support is useful in clinical decision-making; (9) telemedicine is particularly useful during pandemics/system stress; and (10) my patients are generally receptive to telemedicine.

Extended Reality was assessed with the XR Utility Index (XUI; average of 2 items; 1–5 scale), comprising: (X1) XR meaningfully contributes to medical simulation/training, and (X2) XR has strong potential for complex procedures/telementoring. Additional single-item predictors (each 1–5) included: Training Priority (priority for structured AI/XR training), Perceived Future Potential (limited → transformational), and Curricular Integration (support for undergraduate/postgraduate integration). Categorical items documented respondents’ main challenge, perceived benefit, key ethical aspect, reservations, preferred adoption strategy, and clinical specialty (A&ICU, Surgical, Medical, Dentistry).

### 2.4. Outcomes and Variable Construction

The Telemedicine Acceptance Index (TAI) averaged 10 Likert-type items covering perceived impact on care quality, efficiency, diagnostic support, patient engagement, error reduction, recommendation frequency, treatment personalization, decision support, usefulness in pandemics, and patient receptivity (1–5; higher = greater acceptance). Internal consistency was high (Cronbach’s α = 0.86). The XR Utility Index (XUI) averaged two items (perceived contribution of XR to medical simulation/training and perceived potential for complex procedures). Because XUI has two items, we summarized it by mean score and used it primarily as a predictor. Additional single-item predictors included Training Priority (the priority assigned to structured AI/XR upskilling), Perceived Future Potential of AI/XR for the health system, and support for Curriculum Integration (undergraduate/postgraduate). Specialties were grouped a priori as A&ICU, Surgical, Medical, and Dentistry to enable stable comparisons with uneven cell sizes.

### 2.5. Statistical Analysis

We summarized categorical variables as counts (%) and continuous indices as mean ± SD. Given small and unequal group sizes and the ordinal nature of composite indices, between-group comparisons used Kruskal–Wallis tests with exact *p*-values; if any omnibus test had been significant, we planned Dunn’s post hoc comparisons with Benjamini–Hochberg false discovery rate (FDR) control at q = 0.10. Associations among indices and predictors used Spearman’s rank correlation (ρ) with two-sided *p*-values; 95% confidence intervals were obtained by bias-corrected accelerated (BCa) bootstrap where relevant. With *n* = 43, two-sided α = 0.05, we had ~80% power to detect |ρ| ≈ 0.40 in correlations; smaller between-group effects were likely underpowered. Non-significant omnibus tests are interpreted accordingly.

To examine independent predictors of acceptance, we modeled TAI via ordinary least squares (OLS), including XUI, Training Priority, Perceived Future Potential, and specialty indicators (reference = A&ICU). We centered continuous predictors prior to moderation analyses. Model assumptions were assessed via residual-versus-fitted plots and Q–Q inspection. Because mild heteroskedasticity is common with Likert composites, we used HC3 robust standard errors throughout the analysis. Multicollinearity was evaluated with variance-inflation factors (all VIFs ≤ 1.6). Goodness-of-fit was reported as R^2^ and adjusted R^2^.

We probed whether the slope of Training Priority varied by specialty using an interaction model (Training Priority × Specialty Group) with HC3 robust SEs. Simple slopes within each specialty were derived via the delta method using the robust covariance matrix. Finally, we explored a mediation structure with Perceived Future Potential as the exposure (X), XUI as the mediator (M), and TAI as the outcome (Y), adjusting for Training Priority and specialty indicators. Indirect effects were estimated via nonparametric bootstrap with 2000 resamples and percentile 95% CIs; significance required CIs excluding zero. All hypothesis tests were two-sided with α = 0.05. Analyses were conducted in open-source software (R v4.3.2 and Python v3.11.5).

## 3. Results

By percentage, A&ICU comprised 53.5%, Surgical 18.6%, Dentistry 14.0%, and Medical 14.0% of respondents. In this study, telemedicine refers to synchronous/asynchronous clinical encounters, as well as decision support; tele-monitoring (remote physiologic surveillance) is a related but distinct service. A&ICU’s larger share reflects local staffing and technology exposure rather than a study focus limited to tele-monitoring (Table 1).

Physicians most frequently report technical (34.9%) and financial (30.2%) barriers to integrating AI/XR into telemedicine. This aligns with implementation realities: robust connectivity, device procurement, integration with EHR/worklists, and maintenance all carry upfront costs and continuous support needs. A notable 23.3% cited patient acceptance—a reminder that clinician enthusiasm alone is insufficient when patients worry about privacy, automation, or lack of human touch. Echoing this, the leading reservation was potential erosion of the patient–physician interaction (41.9%), followed by data security (23.3%) and diagnostic accuracy (18.6%). The ethical priorities dovetail: clinician accountability (27.9%) and data confidentiality (23.3%) top the list, with informed consent, algorithm transparency, and equitable access also prominent, underscoring a practical ethics frame centered on responsibility, safety, and fairness. Encouragingly, physicians propose highly actionable adoption levers: hands-on workshops (48.8%) and continuing education (20.9%)—exactly the kind of experiential training that can build trust and skills. The perceived benefits—chiefly time efficiency (37.2%) and accessibility (27.9%)—are consistent with telemedicine’s value proposition, while personalization and diagnostic precision remain attractive but secondary gains in this sample (Table 2).

Overall Telemedicine Acceptance Index (TAI) averaged 3.90 ± 0.62, indicating generally favorable attitudes. By specialty group, point estimates ranged from 3.61 ± 1.01 (Surgical) to 4.05 ± 0.50 (Dentistry), with A&ICU at 3.98 ± 0.51 and Medical at 3.80 ± 0.49. Despite visible differences in means, the Kruskal–Wallis test was not significant (*p* = 0.732), suggesting that there are no reliable between-group differences in acceptance at this sample size. The larger dispersion within the Surgical group (SD ≈ 1.01) likely reflects heterogeneity of surgical tele-use-cases—from asynchronous wound reviews to XR-assisted tele-mentoring—leading to diverse personal experiences and comfort levels. Dentistry’s higher mean may reflect frequent remote counseling and follow-up use cases and a strong interest in XR for procedural planning; however, with *n* = 6, this should be interpreted cautiously. The lack of significant differences overall underscores that acceptance determinants may be less about specialty identity and more about cross-cutting beliefs and organizational readiness (e.g., training priority or perceived future potential), as presented in Table 3.

The XR Utility Index averaged 3.86 ± 0.86 overall, suggesting moderate-to-high perceived value. Dentistry showed the highest mean (4.33 ± 0.82), consistent with XR’s relevance to pre-procedural visualization, occlusal analysis, and procedural simulations. A&ICU (3.91 ± 0.79) expressed solid enthusiasm—XR can augment team training (airway management, crisis resource management) and remote guidance. The Medical group (3.50 ± 0.84) and Surgical group (3.62 ± 1.06) were more variable; both likely weigh the operational requirements (hardware, fidelity, workflow fit) against benefits. However, the differences were not statistically significant (Kruskal–Wallis *p* = 0.291), as seen in Table 4.

Telemedicine acceptance correlated strongly with XR Utility (ρ = 0.598, *p* < 0.001), implying that clinicians who see tangible XR value also tend to be more positive overall about AI/XR-enabled telemedicine. Acceptance was also associated with assigning higher priority to training (ρ = 0.455, *p* = 0.002), with believing in the future system-level potential of AI/XR (ρ = 0.584, *p* < 0.001), and with supporting curriculum integration (ρ = 0.352, *p* = 0.021). These relationships sketch a coherent pathway: when physicians anticipate macro-level benefits and receive (or desire) structured upskilling, acceptance rises. XR Utility also tracked with Training Priority (ρ = 0.401, *p* = 0.008), Future Potential (ρ = 0.479, *p* = 0.001), and Curriculum Integration (ρ = 0.303, *p* = 0.049), reinforcing the idea that exposure and education lift perceived utility. Correlations between Training Priority and the other education-related constructs (Future Potential, Curriculum Integration) were positive but modest and marginal (*p* ≈ 0.066), likely reflecting ceiling effects—many respondents already endorse integrating AI/XR into undergraduate curricula (mean 4.44 ± 0.73), as described in Table 5.

The model explained 61.8% of the variance in acceptance (R^2^ = 0.618; adj. R^2^ = 0.554), indicating good explanatory power with a compact predictor set. Two variables emerged as independent predictors: Training Priority (B = 0.20, 95% CI 0.02–0.38, p = 0.030) and Future Potential (B = 0.38, 95% CI 0.18–0.58, p = 0.0005). Each one-point increase (on 1–5) in perceived future potential associates with a +0.38 increase in acceptance (also 1–5 scale), a sizeable effect. Training priority shows a smaller but meaningful increment (+0.20 per point). XR Utility trended positive but was not significant after adjustment (B = 0.14, p = 0.142), suggesting its bivariate association with acceptance is partially mediated by global beliefs about future system fit and the priority physicians assign to upskilling (Table 6).

Table 7 (moderation model) probed whether the association between Training Priority and Telemedicine Acceptance (TAI) varies by specialty while adjusting for Perceived Future Potential and XR Utility, using HC3-robust SEs (*n* = 43). Overall fit was strong (R^2^ = 0.684; adj. R^2^ = 0.597), and Future Potential emerged as an independent predictor (B = 0.383, 95% CI 0.112–0.655, *p* = 0.0057), indicating that, net of other factors, stronger beliefs about system-level value relate to higher acceptance. The main effect of Training Priority, centered, was positive but not significant (B = 0.112, *p* = 0.355), and XR Utility was likewise nonsignificant (B = 0.081, *p* = 0.486). None of the interaction terms reached conventional significance: Training Priority × Surgical (B = 0.266, *p* = 0.211), ×Medical (B = 0.541, *p* = 0.126), ×Dentistry (B = –0.241, *p* = 0.745), and specialty main effects vs. A&ICU were null.

**Figure 1:**
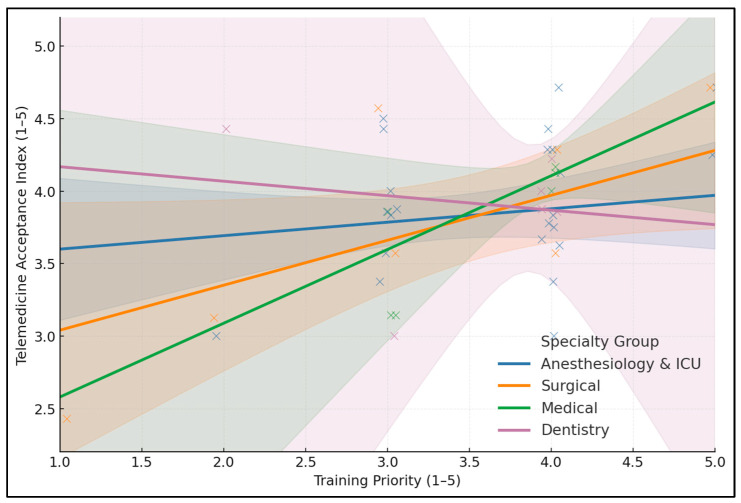
Specialty-specific simple slopes for the association between Training Priority and Telemedicine Acceptance (TAI). Lines show predicted TAI across the 1–5 Training Priority range, adjusted for Future Potential and XUI (HC3 SEs).

In Surgical specialties, each 1-point increase in Training Priority is associated with a +0.378 (SE = 0.184) higher Telemedicine Acceptance Index (*p* = 0.0477); in Medical fields, the slope is +0.653 (SE = 0.349; *p* = 0.0706, trend). Effects are small and non-significant in Anesthesiology and ICU (+0.112, SE = 0.120; *p* = 0.3613) and Dentistry (–0.129, SE = 0.739; *p* = 0.8621), while Future Potential remains an independent positive predictor in the model (B = 0.383, *p* = 0.0057), as presented in Figure 1.

Table 8 shows that a one-point increase in Training Priority corresponds to a significant increase in TAI among Surgical respondents (slope = +0.378, SE = 0.184, *p* = 0.0477), a larger but trend-level increase among Medical respondents (slope = +0.653, SE = 0.349, *p* = 0.0706), a small, nonsignificant increase in A&ICU (slope = +0.112, SE = 0.120, *p* = 0.3613), and a null, imprecise association in Dentistry (slope = –0.129, SE = 0.739, *p* = 0.8621). These within-group estimates are directionally consistent with the hypothesis that training matters most where hands-on, workflow-proximal use cases are salient, such as for surgeons.

The mediation model tested whether XR Utility (XUI) transmits the effect of Perceived Future Potential on acceptance. The “a” path (Future Potential → XUI) was positive (a = 0.406), indicating that clinicians who see greater system-level potential also tend to rate XR as more useful. The “b” path (XUI → TAI | Future Potential) was modest (b = 0.139), and the nonparametric bootstrap showed a small indirect effect (a × b = 0.056) with a 95% CI spanning zero (–0.027 to 0.207), indicating no statistically significant mediation. By contrast, the total effect of Future Potential on TAI was moderate and significant (c = 0.434; 95% CI 0.22–0.62), and the direct effect remained significant after accounting for XUI (c′ = 0.378; 95% CI excluding zero). Approximately 13% of the total effect was attributable to the indirect pathway, reinforcing that beliefs about macro-level potential primarily influence acceptance directly, rather than operating through perceived XR utility (Table 9).

**Figure 2:**
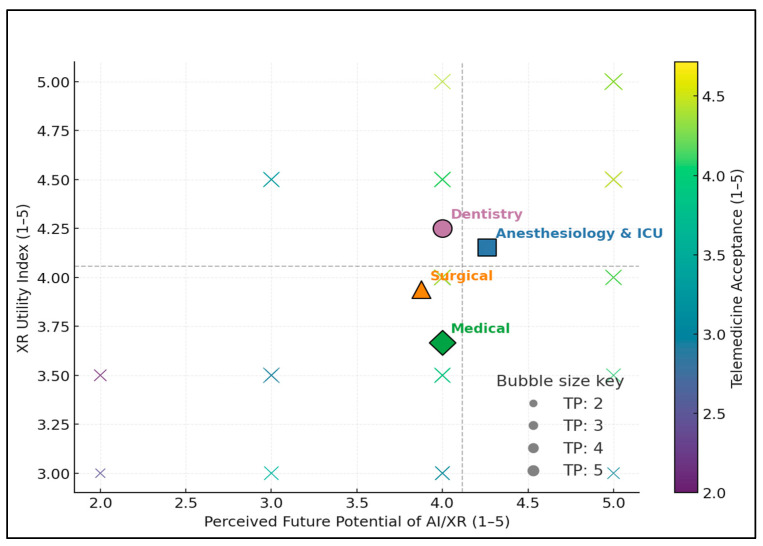
Mediation model testing XR Utility (XUI) as a mediator of Perceived Future Potential → TAI, adjusted for Training Priority and specialty.

Telemedicine acceptance rises with both Future Potential (Spearman ρ = 0.584, *p* < 0.001) and XR Utility (Spearman ρ = 0.598, *p* < 0.001), producing warmer colors toward the upper-right of the plot. Specialty centroids show Dentistry with the highest XR Utility (mean = 4.33), followed by Anesthesiology and ICU (3.91), Surgical (3.62), and Medical (3.50); larger bubbles (higher Training Priority) co-locate with higher acceptance, consistent with the Training Priority–acceptance correlation (ρ = 0.455, *p* = 0.0022), as presented in Figure 2.

## 4. Discussion

### 4.1. Analysis of Findings

This study advances beyond frequency reporting by quantifying predictive pathways for physician acceptance using a compact, theory-anchored framework. Specifically, we demonstrate independent effects of future system value and training priority, test specialty-specific moderation of the training–acceptance slope, and evaluate mediation by XR task-utility, thereby linking practical implementation levers to established acceptance theory.

This multi-specialty sample of Romanian physicians expressed generally favorable attitudes toward AI/XR-enabled telemedicine (TAI = 3.9/5), with especially strong endorsement of integrating these topics into medical curricula (mean = 4.4/5). Importantly, acceptance was not a function of specialty group per se; rather, it tracked with beliefs about future system value and with prioritization of professional training. This pattern underscores that addressing how AI/XR fits within clinical pathways, and equipping clinicians with the skills to evaluate and apply tools safely, may matter more than tailoring messages purely by specialty.

Perceived barriers were concrete: technical reliability, financial outlay, and patient acceptance. Ethical focal points, clinician accountability, and data confidentiality signal a desire for guardrails that preserve professional responsibility and trust. Requested adoption strategies (hands-on workshops and continuing education) map directly onto these needs. XR’s perceived utility was moderate to high across the board and most pronounced in dentistry and A&ICU; however, the differences were not significant, implying that early pilots in receptive services could generate templates others can reuse.

The near-null slopes in A&ICU and Dentistry plausibly reflect ceiling effects (higher baseline familiarity and frequent simulation/remote counseling) and constraint profiles where training alone is not rate-limiting. In A&ICU, ongoing team simulation may already embed AI/XR concepts; in Dentistry, hardware ergonomics, setup time, and reimbursement can dominate perceived feasibility, dampening the marginal yield of generic workshops.

The multivariable model offers a practical blueprint. Acceptance is higher where physicians (a) are convinced of future system-level benefits and (b) view training as a priority. Implementation should therefore pair a compelling, governance-aligned narrative (safety, equity, accountability) with experiential learning (simulations, supervised cases, checklists). This approach addresses both head (evidence, policy) and hands (skills, workflows), while respecting core ethical commitments.

Romanian physicians’ generally favorable views of AI/XR in telemedicine, with acceptance tightly linked to beliefs about future system-level value and the priority given to training, align with several recent clinician surveys outside Romania. A statewide U.S. survey of frontline physicians/physician associates reported broadly positive attitudes toward AI’s potential but highlighted uneven familiarity and a desire for practical education and governance clarity [18]. A national sample of U.S. physicians similarly found limited formal knowledge about AI, coupled with interest in training and clear rules of the road, suggesting that “confidence through competence” is a common precondition for adoption [19]. Scoping evidence across multiple specialties reinforces these patterns, identifying training, workflow fit, and perceived usefulness as recurrent facilitators, with liability and transparency concerns as drag factors [20]. At the pipeline level, Romanian medical students express strong intentions to use digital health yet cite curricular and support needs—consistent with our finding that curriculum integration and training priority co-travel with acceptance among practicing clinicians [21].

Our cross-specialty results also converge with the XR literature. An umbrella review of extended reality (XR) in surgical training concludes that XR is generally acceptable and useful for skill acquisition, while emphasizing the importance of ergonomics, reliability, and instructional design—determinants that mirror the “technical” and “hands-on” themes reported by our respondents [22]. Specialty-specific studies support this: surgeons and residents exposed to augmented reality (AR) report high perceived value and usability when tasks are proximal to operative workflows [23], and orthopedic teams find AR feasible in the operating room, provided setup and visualization are dependable [24]. That our sample’s XR utility scores were strongest in dentistry and A&ICU is consistent with domains where visualization, simulation, and crisis resource management training can benefit from immersive tools, though the literature stresses that stable hardware, network performance, and targeted scenarios are prerequisites for sustained uptake [22,23,24]. However, the absence of a statistically significant indirect effect via XUI suggests that system-level expectations (interoperability, governance, funding) outweigh modality-specific XR beliefs when clinicians form acceptance intentions.

Perceived benefits in our data—time efficiency and improved access—track closely with surgical teleconsultation evidence. A nationwide survey of surgeons reported high interest in video visits for selected indications, but flagged physical exam limitations, rapport challenges, and connectivity as top barriers—the same categories our respondents selected most often [25]. A systematic review comparing online video versus face-to-face surgeon–patient consultations found shorter waiting and total appointment times and broadly similar satisfaction for follow-ups, while calling for more trials in high-stakes preoperative settings—again mapping onto our respondents’ enthusiasm for efficiency and caution about clinical adequacy [26]. At a systems level, an overview of telemedicine reviews across the WHO European Region likewise highlights infrastructure reliability, integration with records, and medico-legal clarity as determinants of sustainable practice—elements our participants prioritized as “technical” and “financial” challenges needing institutional solutions [27,28,29].

Reservations in our sample about erosion of the patient–physician relationship and data confidentiality are also well supported. Comparative and qualitative syntheses describe mixed effects of telehealth on empathy and rapport—some patients and clinicians experience diminished nonverbal connection, while others note unique windows into patients’ home contexts—underscoring that communication training and visit design (e.g., camera placement, agenda-setting, hybrid pathways) materially influence perceived care quality [30,31]. These findings suggest that “acceptance” is inseparable from interaction quality: our observed link between training priority and acceptance is compatible with evidence that targeted upskilling in telecommunication skills can mitigate relational concerns and convert technical feasibility into perceived care value [26,30,31].

Governance signals in our cohort—emphasis on accountability, confidentiality, and transparency—mirror recent policy and institutional developments. Within the EU, the new AI Act establishes a risk-based framework with stringent obligations for high-risk medical AI and interactions with MDR/IVDR, highlighting the need for robust post-market monitoring, documentation, and human oversight—requirements that directly address clinicians’ safety and accountability concerns [28,29]. At the health-system level, case studies describe operational governance for clinical AI (e.g., model review committees, incident reporting, equity monitoring), offering practical templates for aligning frontline training with trustworthy deployment—precisely the blend our respondents favored (hands-on workshops embedded in clear rules) [32]. Together, these developments make “future potential” more credible by reducing uncertainty around responsibility and risk.

The moderation result of the current study is that the training–acceptance slope was most pronounced among surgeons and trended positive in medical specialties, which fits the literature’s lesson that adoption rises when training is proximal to real tasks and when organizational frameworks reduce friction. In practice, this argues for service-line pilots that pair experiential upskilling with concrete governance (XR-supported telementoring with clear data flows and audit trails), while aligning with macro-level regulatory expectations (EU AI Act) and micro-level communication best practices to preserve rapport [28,29,30,31,32]. In short, the external literature supports our core implementation levers: make the future value proposition tangible with guardrails and invest in workflow-proximal training that builds both technical and communicative competence—particularly in specialties where the marginal benefit is highest.

Concurrently, advances in secure medical IoT, including dynamic ciphering schemes designed for healthcare data streams, illustrate practical pathways to harden telemedical ecosystems against interception and tampering [33]. In parallel, digital-twin and embodied-AI developments in mechatronics inform the reliability requirements for XR-enabled tele-operation and telementoring—latency, stability, and safety constraints that clinicians implicitly weigh when judging utility [34].

Practice implications can be specialty-tailored as follows: for Surgical services, prioritize XR-assisted telementoring workshops with OR-adjacent setup drills, overseen by credentialed proctors and standardized checklists that cover visualization quality, network latency, data-capture requirements, and explicit human-oversight points. In A&ICU settings, run crisis-resource management simulations that integrate AI triage/risk scores and structured handoffs, coupled with exercises on fail-safe reversion when data streams degrade. Medical departments should adopt consult-oriented modules on AI-supported triage, differential-diagnosis safety nets, and documentation aids, with clear emphasis on medico-legal boundaries. Dentistry can use XR planning sandboxes focused on occlusion and anatomy, set time-to-setup and ergonomic targets, and define practical pathways for device sharing and sterilization logistics. Cross-cutting across all modules, align activities with local AI governance (model review, incident reporting, equity monitoring) and rigorous data-protection protocols.

Sustainable adoption hinges on (i) reliable broadband and secure device provisioning; (ii) interoperable EHR integration and audit trails; (iii) clear financing for devices/licensing/training; and (iv) alignment with EU risk-based AI regulation (human oversight, documentation, post-market monitoring). Institutional AI governance committees can translate these macro-rules into service-line checklists and approval pathways that clinicians trust.

Beyond descriptive attitudes, this work quantifies predictive pathways for acceptance (training priority and future potential as independent predictors) and tests specialty moderation and mediation by XR utility using robust SEs and bootstrap procedures, thereby advancing theory-anchored implementation levers rather than reporting frequencies alone. Proposed additional XR items (do not affect current results): (i) XR ergonomics are acceptable for routine clinical use; (ii) XR setup time is compatible with clinical workflow; (iii) Network reliability is sufficient for XR telementoring.

The reported acceptance in this cohort aligns with clinician samples from other systems that emphasize utility, governance clarity, and training as recurring determinants of telemedicine and AI uptake. Differences in specialty patterns—stronger training–acceptance coupling in procedure-dense services and more variable effects in ambulatory medical fields—mirror reports from surgical and critical-care settings, whereas medico-legal comfort and workflow fit remain salient in ambulatory care. Together, these consistencies support the external validity of the two actionable levers identified here—credible future system value and structured, workflow-proximal training—and underscore the need to embed hands-on upskilling within clear institutional governance.

### 4.2. Study Limitations

This training-oriented, cross-sectional survey relied on a small, convenience sample (*n* = 43) with uneven specialty strata, over half from A&ICU, which limits precision, interaction power, and generalizability beyond the participating clinicians and Romanian context. Findings reflect Romanian practice patterns and regulatory context and may not generalize to other systems without considering differences in infrastructure, financing, and governance. All measures were self-reported and mapped to 5-point Likert-type scales after translation, introducing potential common-method bias, residual translation artifacts, and untested measurement invariance across specialties. Moreover, we did not measure prior digital training, local IT infrastructure, EHR integration maturity, reimbursement exposure, or medico-legal familiarity; these may confound or moderate observed associations and should be collected prospectively. The TAI composite showed good internal consistency, but the XUI comprised only two items, constraining construct breadth. XUI included two face-valid items to minimize respondent burden; broader XR constructs (ergonomics, cognitive load, workflow fit) warrant multi-item scales and validation (EFA/CFA) in larger samples. Although we used HC3-robust SEs and bootstrap procedures, multiple modeling steps and exploratory interaction probing raise the possibility of type I/II error in a low-power setting. Our design cannot establish causality, and we did not observe actual behavior change or clinical outcomes following training or XR exposure. The convenience sample (*n* = 43) limits precision, particularly for interaction terms and small between-group differences. Future work should employ stratified sampling with pre-specified cell sizes to ensure balanced specialty comparisons and improved generalizability. Self-report Likert data are susceptible to acquiescence and common-method bias. Future work should triangulate with behavioral indicators (e.g., audit logs, credentialing uptake) and vignette-based performance tasks. Finally, several relevant covariates, prior digital training, local infrastructure quality, EHR integration maturity, reimbursement exposure, and medico-legal climate, were not measured and could confound or moderate observed associations.

## 5. Conclusions

Physicians in Romania reported favorable, cross-specialty acceptance of AI/XR-enabled telemedicine. Acceptance was more closely tied to beliefs about future system-level value and prioritized, hands-on training than to specialty identity per se. Implementation should therefore pair governance-aligned messaging (safety, accountability, data protection) with experiential upskilling (workshops, supervised use cases) embedded in clinical pathways, starting where marginal benefit is highest (surgical telementoring, A&ICU simulation). Future studies should employ probabilistic sampling, broaden XR construct measurement, and link attitudinal shifts to behavioral adoption and patient outcomes in longitudinal designs.

## Figures and Tables

**Table 1:** Specialty distribution (*n* = 43).

Specialty	*n*	%
Anesthesiology and Intensive Care	23	53.5
Dentistry	6	14
Plastic surgery and reconstructive microsurgery	4	9.3
Neurosurgery	4	9.3
Neurology	3	7
Rehabilitation and Physical Medicine	1	2.3
Gastroenterology	1	2.3
Endocrinology	1	2.3

Telemedicine here denotes clinical encounters and decision support; tele-monitoring refers to remote physiologic surveillance. Sampling prioritized practicing clinicians across services; A&ICU over-representation mirrors local staffing/exposure rather than a tele-monitoring focus.

**Table 2:** Professional context and perceived practice needs.

Domain	Category	*n*	%
Challenge	Technical	15	34.9
Challenge	Financial	13	30.2
Challenge	Patient acceptance	10	23.3
Challenge	Staff training	5	11.6
Reservation	Patient–physician interaction	18	41.9
Reservation	Data security	10	23.3
Reservation	Diagnostic accuracy	8	18.6
Reservation	Patient acceptance	5	11.6
Reservation	Costs	2	4.7
Ethical aspect	Clinician accountability	12	27.9
Ethical aspect	Data confidentiality	10	23.3
Ethical aspect	Informed consent	9	20.9
Ethical aspect	Algorithm transparency	6	14
Ethical aspect	Equity of access to care	6	14
Adoption strategy	Hands-on demonstrations and workshops	21	48.8
Adoption strategy	Continuing training and education	9	20.9
Adoption strategy	Partnerships between healthcare and tech sectors	6	14
Adoption strategy	Financial support and resources	5	11.6
Adoption strategy	Innovation culture in healthcare	2	4.7
Perceived benefit	Time efficiency	16	37.2
Perceived benefit	Increased accessibility	12	27.9
Perceived benefit	Improved treatment personalization	8	18.6
Perceived benefit	Improved diagnostic accuracy	7	16.3

**Table 3:** Telemedicine acceptance by specialty group.

Specialty Group	*n*	Mean	SD	Kruskal–Wallis *p*
Anesthesiology and ICU	23	3.98	0.51	0.7324
Dentistry	6	4.05	0.5	0.7324
Medical	6	3.8	0.49	0.7324
Surgical	8	3.61	1.01	0.7324

**Table 4:** XR utility by specialty group.

Specialty Group	*n*	Mean	SD	Kruskal–Wallis *p*
Anesthesiology and ICU	23	3.91	0.79	0.2912
Dentistry	6	4.33	0.82	0.2912
Medical	6	3.5	0.84	0.2912
Surgical	8	3.62	1.06	0.2912

**Table 5:** Spearman correlations among key indices (*n* = 43).

Var1	Var2	Spearman_Rho	*p*_Value
Telemedicine_Acceptance_Index	XR_Utility_Index	0.598	0
Telemedicine_Acceptance_Index	Training_Priority	0.455	0.0022
Telemedicine_Acceptance_Index	Future_Potential	0.584	0
Telemedicine_Acceptance_Index	Curriculum_Integration	0.352	0.0206
XR_Utility_Index	Training_Priority	0.401	0.0078
XR_Utility_Index	Future_Potential	0.479	0.0012
XR_Utility_Index	Curriculum_Integration	0.303	0.0485
Training_Priority	Future_Potential	0.283	0.0658
Training_Priority	Curriculum_Integration	0.282	0.0667
Future_Potential	Curriculum_Integration	0.472	0.0014

**Table 6:** Multivariable model predicting telemedicine acceptance.

Predictor	B	CI_Low	CI_High	*p*
Intercept	1.095	0.264	1.925	0.0113
XR_Utility_Index	0.139	−0.049	0.326	0.1424
Training_Priority	0.202	0.021	0.382	0.03
Future_Potential	0.378	0.179	0.577	0.0005
Specialty_Group_Surgical	−0.053	−0.415	0.309	0.768
Specialty_Group_Medical	0.004	−0.388	0.396	0.9841
Specialty_Group_Dentistry	0.138	−0.265	0.541	0.4908

Model fit: R^2^ = 0.684; adj. R^2^ = 0.597 (*n* = 43; HC3 robust SEs); Standardized coefficients (β) computed from z-scored variables: Future Potential β ≈ 0.52; Training Priority β ≈ 0.27; XR Utility β ≈ 0.18.

**Table 7:** Moderation model: Does the effect of Training Priority on Telemedicine Acceptance vary by Specialty Group?

Predictor	B	SE	CI_Low	CI_High	*p*
Intercept	3.905	0.089	3.73	4.079	0
Training_Priority (centered)	0.112	0.12	−0.125	0.348	0.3546
Future_Potential (centered)	0.383	0.139	0.112	0.655	0.0057
XR_Utility_Index (centered)	0.081	0.116	−0.146	0.307	0.4863
Training_Priority × Surgical	0.266	0.213	−0.151	0.683	0.2105
Training_Priority × Medical	0.541	0.354	−0.152	1.235	0.1261
Training_Priority × Dentistry	−0.241	0.739	−1.69	1.208	0.7446
Surgical (vs. A&ICU)	0.004	0.199	−0.386	0.393	0.9849
Medical (vs. A&ICU)	−0.039	0.186	−0.404	0.326	0.8353
Dentistry (vs. A&ICU)	0.153	0.361	−0.554	0.86	0.671

Model fit: R^2^ = 0.684; adj. R^2^ = 0.597 (*n* = 43; HC3 robust SEs).

**Table 8:** Simple slopes of Training Priority (per 1-point) by specialty (delta method; df = mod df_resid).

Specialty_Group	Slope (Per 1-Point Training Priority)	SE	*p*
Anesthesiology and ICU	0.112	0.12	0.3613
Surgical	0.378	0.184	0.0477
Medical	0.653	0.349	0.0706
Dentistry	−0.129	0.739	0.8621

**Table 9:** Mediation analysis (2000 bootstrap resamples).

Effect	Estimate	Boot 95% CI (Lower)	Boot 95% CI (Upper)
a (X → M)	0.406	—	—
b (M → Y	X)	0.139	—
c (total X → Y)	0.434	0.22	0.62
c’ (direct X → Y	M)	0.378	0.119
Indirect (a × b)	0.056	−0.027	0.207
Proportion mediated	0.13	—	—

## Data Availability

The data presented in this study are available on reasonable request from the corresponding author; the data are not publicly available due to patient privacy and ethical restrictions.

## References

[1] Tuckson R.V., Edmunds M., Hodgkins M.L. (2017). Telehealth. N. Engl. J. Med..

[2] Koonin L.M., Hoots B., Tsang C.A., Leroy Z., Farris K., Jolly T., Antall P., McCabe B., Zelis C.B.R., Tong I. (2020). Trends in the Use of Telehealth During the Emergence of the COVID-19 Pandemic—United States, January–March 2020. MMWR Morb. Mortal. Wkly. Rep..

[3] Kuan P.X., Chan W.K., Fern Ying D.K., Rahman M.A.A., Peariasamy K.M., Lai N.M., Mills N.L., Anand A. (2022). Efficacy of telemedicine for the management of cardiovascular disease: A systematic review and meta-analysis. Lancet Digit. Health.

[4] Pereira A.J., Noritomi D.T., Dos Santos M.C., Corrêa T.D., Ferraz L.J.R., Schettino G.P.P., Cordioli E., Morbeck R.A., Morais L.C., Salluh J.I.F. (2024). Effect of Tele-ICU on Clinical Outcomes of Critically Ill Patients: The TELESCOPE Randomized Clinical Trial. JAMA.

[5] Holden R.J., Karsh B.T. (2010). The technology acceptance model: Its past and its future in health care. J. Biomed. Inform..

[6] Diel S., Doctor E., Reith R., Buck C., Eymann T. (2023). Examining supporting and constraining factors of physicians’ acceptance of telemedical online consultations: A survey study. BMC Health Serv. Res..

[7] Bîlbîie A., Puiu A.I., Mihăilă V., Burcea M. (2024). Investigating Physicians’ Adoption of Telemedicine in Romania Using Technology Acceptance Model (TAM). Healthcare.

[8] Rajkomar A., Dean J., Kohane I. (2019). Machine Learning in Medicine. N. Engl. J. Med..

[9] Obermeyer Z., Powers B., Vogeli C., Mullainathan S. (2019). Dissecting racial bias in an algorithm used to manage the health of populations. Science.

[10] Ghassemi M., Oakden-Rayner L., Beam A.L. (2021). The false hope of current approaches to explainable artificial intelligence in health care. Lancet Digit. Health.

[11] Alanazi A.T. (2023). Clinicians’ Perspectives on Healthcare Cybersecurity and Cyber Threats. Cureus.

[12] Vera A.M., Russo M., Mohsin A., Tsuda S. (2014). Augmented reality telementoring (ART) platform: A randomized controlled trial to assess the efficacy of a new surgical education technology. Surg. Endosc..

[13] Wild C., Lang F., Gerhäuser A.S., Schmidt M.W., Kowalewski K.F., Petersen J., Kenngott H.G., Müller-Stich B.P., Nickel F. (2022). Telestration with augmented reality for visual presentation of intraoperative target structures in minimally invasive surgery: A randomized controlled study. Surg. Endosc..

[14] Lee A.T., Ramasamy R.K., Subbarao A. (2025). Understanding Psychosocial Barriers to Healthcare Technology Adoption: A Review of TAM Technology Acceptance Model and Unified Theory of Acceptance and Use of Technology and UTAUT Frameworks. Healthcare.

[15] Petre I., Barna F., Gurgus D., Tomescu L.C., Apostol A., Petre I., Furau C., Năchescu M.L., Bordianu A. (2023). Analysis of the Healthcare System in Romania: A Brief Review. Healthcare.

[16] Abbas Q., Jeong W., Lee S.W. (2025). Explainable AI in Clinical Decision Support Systems: A Meta-Analysis of Methods, Applications, and Usability Challenges. Healthcare.

[17] Yu F., Iu H.-C., Lin H., Pham V.-T. (2025). Editorial: Advances in Nonlinear Systems and Networks, Volume III. Front. Phys..

[18] Dean T.B., Seecheran R., Badgett R.G., Zackula R., Symons J. (2024). Perceptions and attitudes toward artificial intelligence among frontline physicians and physicians’ assistants in Kansas: A cross-sectional survey. JAMIA Open.

[19] Bajwa J., Munir U., Nori A., Williams B. (2021). Artificial intelligence in healthcare: Transforming the practice of medicine. Future Healthc. J..

[20] Scipion C.E.A., Manchester M.A., Federman A., Wang Y., Arias J.J. (2025). Barriers to and facilitators of clinician acceptance and use of artificial intelligence in healthcare settings: A scoping review. BMJ Open.

[21] Lotrean L.M., Sabo S.A. (2023). Digital Health Training, Attitudes and Intentions to Use It among Romanian Medical Students: A Study Performed during COVID-19 Pandemic. Healthcare.

[22] Toni E., Toni E., Fereidooni M., Ayatollahi H. (2024). Acceptance and use of extended reality in surgical training: An umbrella review. Syst. Rev..

[23] Ramalhinho J., Yoo S., Dowrick T., Koo B., Somasundaram M., Gurusamy K., Hawkes D.J., Davidson B., Blandford A., Clarkson M.J. (2023). The value of Augmented Reality in surgery—A usability study on laparoscopic liver surgery. Med. Image Anal..

[24] Canton S.P., Austin C.N., Steuer F., Dadi S., Sharma N., Kass N.M., Fogg D., Clayton E., Cunningham O., Scott D. (2024). Feasibility and Usability of Augmented Reality Technology in the Orthopaedic Operating Room. Curr. Rev. Musculoskelet. Med..

[25] Kulkarni A.J., Thiagarajan A.B., Skolarus T.A., Krein S.L., Ellimoottil C. (2024). Attitudes and barriers toward video visits in surgical care: Insights from a nationwide survey among surgeons. Surgery.

[26] Ten Haaft B.H.E.A., Montorsi R.M., Barsom E., Kazemier G., Schijven M.P., Besselink M.G. (2024). Online video versus face-to-face patient-surgeon consultation: A systematic review. Surg. Endosc..

[27] Saigí-Rubió F., Borges do Nascimento I.J., Robles N., Ivanovska K., Katz C., Azzopardi-Muscat N., Novillo Ortiz D. (2022). The Current Status of Telemedicine Technology Use Across the World Health Organization European Region: An Overview of Systematic Reviews. J. Med. Internet Res..

[28] Schmidt J., Schutte N.M., Buttigieg S., Novillo-Ortiz D., Sutherland E., Anderson M., de Witte B., Peolsson M., Unim B., Pavlova M. (2024). Mapping the regulatory landscape for artificial intelligence in health within the European Union. npj Digit. Med..

[29] Aboy M., Minssen T., Vayena E. (2024). Navigating the EU AI Act: Implications for regulated digital medical products. npj Digit. Med..

[30] Jabour A.M. (2024). A comparative study of patient-physician empathy in telehealth and traditional in-person visits. Digit. Health.

[31] Andreadis K., Muellers K., Ancker J.S., Horowitz C., Kaushal R., Lin J.J. (2023). Telemedicine Impact on the Patient-Provider Relationship in Primary Care During the COVID-19 Pandemic. Med. Care.

[32] Saenz A.D., Centi A., Ting D., You J.G., Landman A., Mishuris R.G., Mass General Brigham AI Governance Committee (2024). Establishing responsible use of AI guidelines: A comprehensive case study for healthcare institutions. npj Digit. Med..

[33] Jin J., Wu M., Ouyang A., Li K., Chen C. (2025). A Novel Dynamic Hill Cipher and Its Applications on Medical IoT. IEEE Internet Things J..

[34] Liu Y., Chen W., Bai Y., Liang X., Li G., Gao W., Lin L. (2025). Aligning Cyberspace with the Physical World: A Comprehensive Survey on Embodied AI. IEEE/ASME Trans. Mechatron..

